# Correlations between Brain Cortical Thickness and Cutaneous Pain Thresholds Are Atypical in Adults with Migraine

**DOI:** 10.1371/journal.pone.0099791

**Published:** 2014-06-16

**Authors:** Todd J. Schwedt, Catherine D. Chong

**Affiliations:** Mayo Clinic, Department of Neurology, Phoenix, Arizona, United States of America; University Medical Center Goettingen, Germany

## Abstract

**Background/Objective:**

Migraineurs have atypical pain processing, increased expectations for pain, and hypervigilance for pain. Recent studies identified correlations between brain structure and pain sensation in healthy adults. The objective of this study was to compare cortical thickness-to-pain threshold correlations in migraineurs to healthy controls. We hypothesized that migraineurs would have aberrant relationships between the anatomical neurocorrelates of pain processing and pain thresholds.

**Methods:**

Pain thresholds to cutaneously applied heat were determined for 31 adult migraineurs and 32 healthy controls. Cortical thickness was determined from magnetic resonance imaging T1-weighted sequences. Regional cortical thickness-to-pain threshold correlations were determined for migraineurs and controls separately using a general linear model whole brain vertex-wise analysis. A pain threshold-by-group interaction analysis was then conducted to estimate regions where migraineurs show alterations in the pain threshold-to-cortical thickness correlations relative to healthy controls.

**Results:**

Controls had *negative* correlations (p<0.01 uncorrected) between pain thresholds and cortical thickness in left posterior cingulate/precuneus, right superior temporal, right inferior parietal, and left inferior temporal regions, and a *negative* correlation (p<0.01 Monte Carlo corrected) with a left superior temporal/inferior parietal region. Migraineurs had *positive* correlations (p<0.01 uncorrected) between pain thresholds and cortical thickness in left superior temporal/inferior parietal, right precuneus, right superior temporal/inferior parietal, and left inferior parietal regions. Cortical thickness-to-pain threshold correlations differed between migraine and control groups (p<0.01 uncorrected) for right superior temporal/inferior parietal, right precentral, left posterior cingulate/precuneus, and right inferior parietal regions and (p<0.01 Monte Carlo corrected) for a left superior temporal/inferior parietal region.

**Conclusions:**

Unlike healthy control subjects who have a significant negative correlation between cortical thickness in a superior temporal/inferior parietal region with pain thresholds, migraineurs have a non-significant positive correlation between cortical thickness in a superior temporal/inferior parietal region with pain thresholds. Since this region participates in orienting and attention to painful stimuli, absence of the normal correlation might represent a migraineurs inability to inhibit pain sensation via shifting attention away from the painful stimulus.

## Introduction

Migraineurs have atypical processing of sensory stimuli, including painful stimuli, during and between migraine attacks. During a migraine attack, the majority of migraineurs are hypersensitive to visual, auditory, olfactory and somatosensory stimuli, leading to photophobia, phonophobia, olfactory hypersensitivity, and cutaneous allodynia. [1], [2], [3], [4], [5], [6] Between migraine attacks, a proportion of migraineurs have persistent, although often less prominent, hypersensitivities. [7], [8], [9], [10], [11] Migraineurs also have increased expectations for pain and are hypervigilant regarding future pain. [12], [13]

Imaging experiments investigating brain *function* are beginning to elucidate the mechanisms for migraine hypersensitivities. Several migraine functional magnetic resonance imaging (fMRI) studies have demonstrated hyperexcitability of regions that facilitate sensory processing in response to pain, visual stimuli and olfactory stimuli. [14], [15], [16], [17], [18], [19], [20] However, despite recent evidence of correlations between brain *structure* (e.g. cortical thickness) and pain sensation in healthy people, data investigating correlations between migraine brain structure and pain sensation are lacking from the migraine literature. [21] The objective of this study was to further investigate pain-processing regions in the migraine brain by comparing correlations between cortical thickness and pain thresholds in migraineurs to those in healthy controls. This study aimed to interrogate neuro-anatomical correlates of pain processing in migraineurs relative to healthy controls. We hypothesized that migraineurs would demonstrate atypical cortical thickness-to-pain threshold correlations. Identification of brain regions with atypical cortical thickness-to-pain threshold correlations could elucidate anatomical regions involved in aberrant pain processing, a potentially fruitful step for further defining mechanisms of atypical pain processing by the migraine brain, identifying migraine biomarkers, and identifying brain targets for migraine treatments.

## Methods

### Subjects

After Institutional Review Board approval, 31 consenting adult migraine subjects and 32 healthy control subjects were enrolled into this study. Subjects were 18-65 years of age, did not have contraindications to MRI scanning, did not have contraindications to or disorders that might directly affect measurement of cutaneous pain thresholds (e.g. peripheral neuropathy), did not have acute or chronic pain disorders other than migraine, and did not have other neurological disorders. All subjects had normal brain MRI scans according to routine clinical interpretation. Migraine was diagnosed according to International Classification of Headache Disorders II diagnostic criteria. [22] Migraineurs were free from migraine prophylactic medications and they were not overusing abortive migraine medications. Control subjects did not have headaches other than infrequent tension-type headache (<3 headache days/month). Migraine and control subjects were recruited via advertisements placed in the hospital (e.g. posters and flyers), via word-of-mouth referrals, and from a database of research volunteers maintained by the medical school.

### Testing

All study testing was conducted during one visit. All subjects were free of pain for at least 48 hours and free of pain medications and migraine medications for at least 48 hours. Subjects first completed questionnaires, then had quantitative sensory testing (QST) for determination of pain thresholds, and then underwent magnetic resonance imaging (MRI).

Structured interviews, the State-Trait Anxiety Inventory (STAI, Form Y-1 and Form Y-2), the Beck Depression Inventory (BDI-II), and the Allodynia Symptom Checklist 12 were utilized for determining the presence or absence of migraine, migraine frequency, number of years with migraine, presence of migraine aura, anxiety scores, depression scores, and cutaneous allodynia severity. [23], [24], [25], [26]

QST was used to determine pain thresholds to heat applied to the skin using a 30 mm × 30 mm thermode attached to a Medoc Pathway QST machine. Left ventral medial forearm pain thresholds were determined using the method of limits with a temperature increase of 1°C per second. Testing was performed 3 times and the mean of the 3 tests was considered the pain threshold.

All neuroimaging was performed on a Siemens MAGNETOM Trio 3 tesla scanner (Erlangen, Germany) with total imaging matrix technology using a 12-channel head matrix coil and FDA-approved sequences. Structural scans included a high-resolution T1-weighted sagittal magnetization-prepared rapid gradient echo (MP-RAGE) (TE  =  3.16 ms, TR  =  2.4 s, flip angle  =  8°, 176 slices, 1 × 1 × 1 mm voxels, field-of-view 256 × 256 mm^2^) and an axial T2-weighted turbo spin echo (TE  =  88 ms, TR  =  6280 ms, flip angle  =  120°, 36 slices, 1 × 1 × 4 mm voxels, field-of-view 256 × 256 mm^2^).

### MRI Data Processing and Analyses

For cortical reconstruction and segmentation, T1-weighted MP-RAGE sequences were processed using FreeSurfer (http://surfer.nmr.mgh.harvard.edu). All image post-processing was performed on a single Mac workstation running OS X Lion 10.7.5 software by a single technician. Processing utilized methods that have been described in detail in prior publications, including skull stripping, automated Talairach transformation, segmentation of subcortical white matter and gray matter structures, intensity normalization, tessellation of brain boundaries, automated topology correction, and surface deformation. [27], [28], [29], [30], [31], [32], [33], [34], [35], [36], [37], [38], [39] FreeSurfer output was visually inspected for errors prior to data being included for further analyses. Cortical thickness was defined as the distance from the boundary of the gray matter with white matter to the boundary of the gray matter with cerebrospinal fluid at each vertex along the brain surface. [29] For data interpretation and analysis, a surface-based toolbox within FreeSurfer was used to interrogate cortical thickness to pain threshold correlations in migraineurs and healthy controls. Cortical thickness output maps were smoothed by applying a smoothing kernel of 20 mm FWHM (full width at half maximum).

### Statistical Analyses

Subject demographics, pain thresholds, total cortical thickness, cutaneous allodynia severity scores, anxiety and depression scores were reported using descriptive statistics and compared amongst subject groups using independent sample t-tests (two-tailed) or chi-squared tests, as appropriate. Cortical thickness-to-pain threshold correlations were analyzed separately for migraine subjects and for control subjects. Correlations were analyzed by applying a general linear model (GLM) whole brain vertex-wise analysis within FreeSurfer. Age, Beck Depression Inventory scores, trait and state anxiety scores, and estimated total intracranial volume were entered into the GLM as covariates of no interest. Cortical thickness-to-pain threshold correlations surviving p<.01 uncorrected and those surviving Monte Carlo cluster correction of p<.01 were determined for migraine and control groups separately. [40] Next, a pain threshold-by-group interaction analysis was conducted to determine if there were differences between migraine and controls subjects in cortical regions that correlate with pain thresholds. Comparisons surviving p<0.01 uncorrected and those surviving Monte Carlo cluster correction of p<0.01 were identified.

In a post-hoc analysis, the mean cortical thickness of regions that had correlations with pain thresholds that differed in migraineurs vs. controls and survived Monte Carlo cluster correction with p<0.01 were calculated and exported into SPSS 22.0 (SPSS Inc., Chicago, IL USA). Mean cortical thickness of these regions in migraineurs was compared to mean cortical thickness in controls using independent sample t-tests. Correlations between cortical thickness in these regions and headache frequency, number of years with migraine, and allodynia scores were calculated for the migraineurs. In these post-hoc analyses, comparisons and correlations with p<.05 were considered significant.

## Results

### Subjects (Table 1)

**Table 1:** Subject Characteristics.

	Migraine (n = 31)	Control (n = 32)	p-value
**Age** (years)	34.9 (12.1)	35.3 (11.6)	.88
**Gender** (F/M)	26/5	25/7	.57
**State Anxiety Score**	25.4 (6.1)	25.2 (5.8)	.88
**Trait Anxiety Score**	31.0 (9.9)	29.4 (9.3)	.51
**Depression Score**	4.8 (5.0)	2.8 (4.9)	.12
**Allodynia Score**	.52 (1.6)	.13 (.34)	.19
**Pain Threshold**	43.5°C (3.7)	43.7°C (3.4)	.81
**Headache Frequency** (days/month)	7.8 (5.8)	NA	NA
**Years with Migraine**	16.0 (9.2)	NA	NA
**Migraine Aura (yes/no)**	10/21	NA	NA

All continuous variables are reported as means with standard deviation in parentheses. Categorical variables are reported as counts. State and Trait anxiety was determined via the Spielberger State-Trait Anxiety questionnaire. Depression scores were determined via the Beck Depression Inventory (BDI-II). Allodynia scores were determined via the Allodynia Symptom Checklist 12.

Average age was 34.9 years (+/− 12.1) in the migraineurs (n = 31) and 35.3 years (+/− 11.6) in the controls (n = 32). Approximately 80% of all subjects were women, consistent with the predominance of migraine amongst women. There were no significant differences in age, gender distribution, anxiety scores, or depression scores between the migraine and control groups. Mean pain threshold and allodynia symptom severity scores did not differ between the two subject cohorts. There were no significant differences between groups for total cortical thickness over both hemispheres (migraineurs 2.48 mm vs. controls 2.51 mm, p  =  .35). Migraineurs averaged 7.8 days with headache per month (+/− 5.8) and had migraine for 16 years (+/− 9.2). Ten migraineurs (32.3% of all migraineurs) had migraine with aura attacks.

### Cortical Thickness Correlations with Pain Thresholds – Migraine vs. Controls (Table 2, Figure 1)

**Figure 1:**
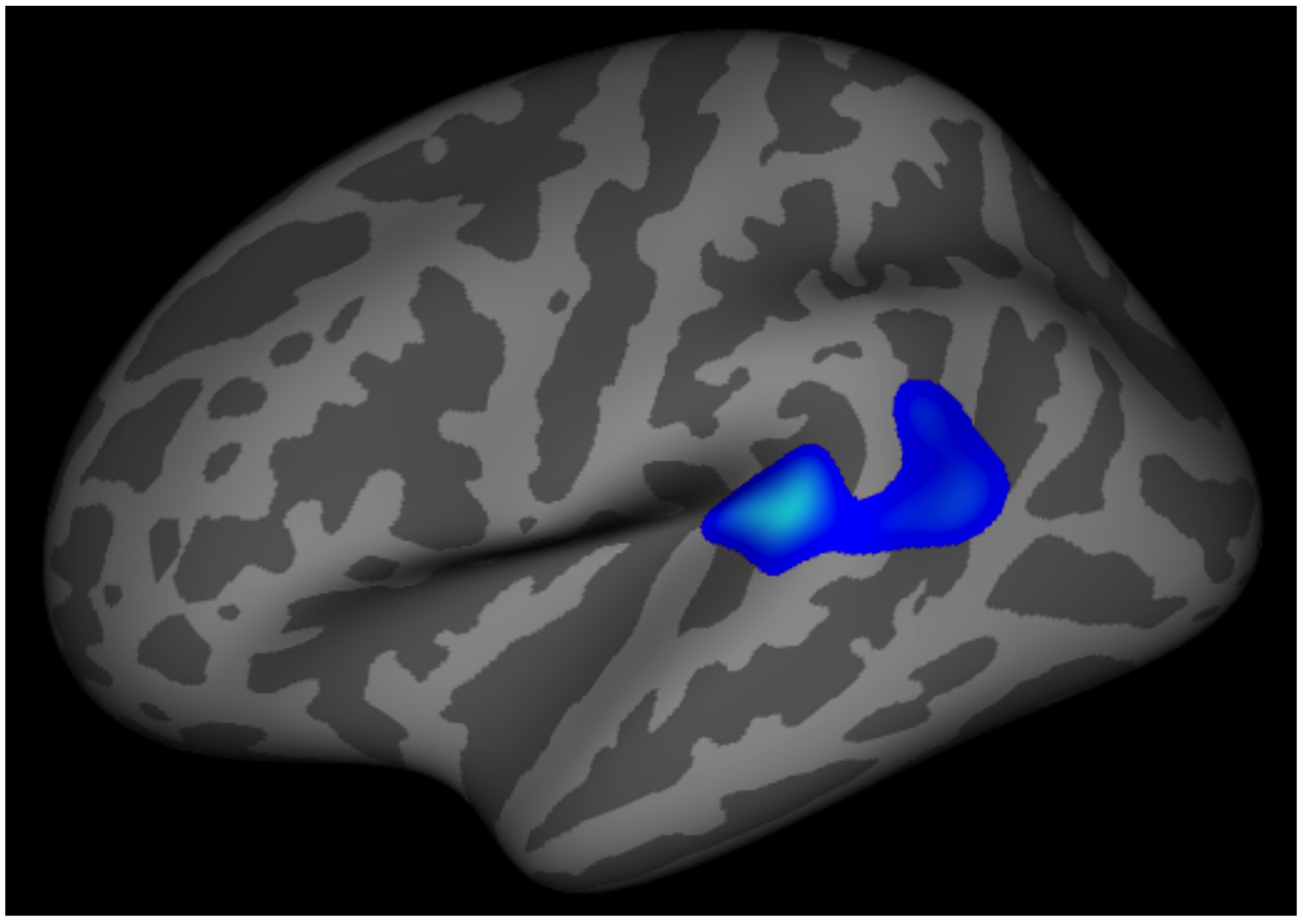
Differences in Cortical Thickness-to-Pain Threshold Correlations between Migraineurs and Controls. Correlation between the cortical thickness of a left superior temporal/inferior parietal region with pain thresholds differed in healthy control subjects vs. migraine subjects. Healthy control subjects demonstrated the expected negative correlation between pain thresholds and cortical thickness in this region while migraineurs lacked this negative correlation.

**Table 2:** Cortical Thickness-to-Pain Threshold Correlations.

	Cortical Region	Region Size (mm^2^)	X	Y	Z
	Left Superior Temporal/Inferior Parietal	412	−43	−39	12
**Migraine**	Right Precuneus	47	7	−64	55
	Right Superior Temporal/Inferior Parietal	39	60	−42	16
	Left Inferior Parietal	14	−43	−54	10
	Left Superior Temporal/Inferior Parietal*	**1542**	**−57**	**−52**	**26**
	Left Posterior Cingulate/Precuneus	224	−17	−29	37
**Control**	Right Superior Temporal	176	59	−35	6
	Right Inferior Parietal	152	32	−77	16
	Left Inferior Temporal	66	−52	−34	−19
	Left Inferior Temporal	10	−44	−42	−17
	Left Superior Temporal/Inferior Parietal*	**1669**	**−47**	**−37**	**13**
**Migraine**	Right Superior Temporal/Inferior Parietal	484	61	−40	15
**vs.**	Right Precentral	248	58	0	33
**Control**	Left Posterior Cingulate/Precuneus	105	−11	−37	41
	Right Inferior Parietal	8	32	−74	17

Correlations between cortical thickness and pain thresholds in migraineurs and controls are listed, as well as those correlations that differed when comparing the migraineurs to controls. All correlations in this table had uncorrected p<.01, while those bolded and starred survived Monte Carlo correction (p<.01). X, Y, and Z coordinates are based on the Talairach and Tournoux Atlas.

In healthy controls there were *negative* correlations between pain thresholds and cortical thickness in left superior temporal/inferior parietal, left posterior cingulate/precuneus, right superior temporal, right inferior parietal, and left inferior temporal regions. Thus, control subjects with lower pain thresholds (i.e. those in whom less intense heat was required to cause pain) had thicker cortex in these regions. The only cortical thickness-to-pain threshold correlation that survived Monte Carlo correction was for the left superior temporal/inferior parietal region.

In migraineurs, there were *positive* correlations between pain thresholds and cortical thickness in left superior temporal/inferior parietal, right precuneus, right superior temporal/inferior parietal, and left inferior parietal regions. Thus, migraine subjects with lower pain thresholds (i.e. were more sensitive to heat pain) had thinner cortex in these regions. However, none of these regions survived Monte Carlo correction.

Cortical thickness to pain threshold correlations differed (p<.01) between migraineurs and controls for left superior temporal/inferior parietal, right superior temporal/inferior parietal, right precentral, left posterior cingulate/precuneus, and right inferior parietal regions. Cortical thickness to pain threshold correlations with the left superior temporal/inferior parietal region were the only correlations that significantly differed between migraineurs and controls after Monte Carlo correction with p<.01. For this region, healthy controls had a significant negative correlation between cortical thickness and pain thresholds while migraineurs had a non-significant positive correlation between cortical thickness and pain thresholds.

Although cortical thickness in this superior temporal/inferior parietal cortex was numerically thinner in migraineurs compared to controls, there was no significant difference in cortical thickness of this region between subject cohorts (2.35 mm +/− 0.39 mm in migraineurs vs. 2.52 mm +/− 0.32 mm in controls, p  =  .071).

Amongst the migraineurs, there were no significant correlations between cortical thickness in the left superior temporal/inferior parietal region and headache frequency, number of years with migraine, or allodynia symptom severity.

## Discussion

The main finding of this study is that migraineurs lacked the normal correlation between cortical thickness of a left superior temporal/inferior parietal region and cutaneous pain thresholds (migraineurs had a non-significant positive correlation), while healthy controls demonstrated the expected negative correlation. Amongst migraineurs, superior temporal/inferior parietal cortical thickness did not correlate with headache frequency, number of years with migraine, or allodynia symptom severity suggesting that the lack of cortical thickness-to-pain threshold correlation might be an underlying trait of the migraine brain as opposed to being a result of recurrent migraine attacks.

### Cortical Thickness and Pain

A study by Erpelding and colleagues published in 2012 was reportedly the first to investigate correlations between cortical thickness and thermal pain thresholds. [21] Subjects were 80 healthy adults aged 19–36 years. Methods of determining pain thresholds and determining cortical thickness were very similar to the methods used in our study reported herein. However, an important difference is that the Erpelding study analysis was restricted to a mask that contained primary somatosensory cortex, secondary somatosensory cortex, paracentral lobule, anterior cingulate cortex, middle cingulate cortex, insular cortex, and prefrontal cortex, while our analyses investigated regions covering the whole brain cortex. In the Erpelding study, there were negative correlations between heat pain thresholds and cortical thickness in regions of the left primary somatosensory cortex, left posterior middle cingulate cortex, and right orbitofrontal cortex. In our study reported herein, negative correlations between the posterior cingulate and heat pain thresholds were also found, but did not survive Monte Carlo correction. Correlations between heat pain thresholds with primary somatosensory cortex and with orbitofrontal cortex were absent, perhaps relating to differences in our study population (e.g. smaller sample size) and use of a whole brain analysis approach.

Correlations between pain sensitivity (i.e. individual subject pain intensity ratings in response to 49 degrees Celsius stimulation of the skin) and regional gray matter density were studied in 116 healthy volunteers. [41] Using voxel-based morphometry (VBM) analyses, investigators found negative correlations between pain intensity ratings and gray matter density of inferior parietal lobule, intraparietal sulcus, precuneus, posterior cingulate cortex and primary sensory cortex. In our study reported herein, negative correlations between inferior parietal lobe cortical thickness and pain thresholds and between posterior cingulate/precuneus cortical thickness and pain thresholds were identified. Although we did not found find correlations for the other regions reported in the Emerson study, numerous factors could explain the different findings including that measuring cortical thickness is not the same as measuring gray matter density, studying pain *intensity* ratings is different than studying thermal pain *thresholds*, and differing sample sizes.

The underlying mechanism by which cortical thickness correlates with pain thresholds is yet to be completely understood. However, several studies have found associations between the presence of pain disorders and atypical cortical thickness, including several migraine studies. [42], [43], [44], [45], [46] It is possible that aberrant cortical thickness of pain sensing and pain modulating regions determines sensitivity to somatosensory stimuli and thus predefines a person's pain perception in terms of pain thresholds, pain intensity and/or pain unpleasantness. [47] Alternatively, recurrent pain might lead to alterations in cortical thickness. Supporting this theory, a longitudinal experiment showed that repetitive painful stimuli delivered over 8 days leads to prolonged but reversible changes in gray matter volume of somatosensory cortex, middle cingulate cortex, and parietal lobe. [48] Other studies have demonstrated that atypical gray matter volume in patients with clinical pain normalizes after pain resolution. [49], [50], [51] Thus, although the mechanisms by which cortical thickness is associated with pain and pain thresholds are yet to be completely elucidated, there is strong evidence that the structure of pain processing regions does in fact associate with clinical and experimental pain.

### Superior Temporal/Inferior Parietal Region and Pain

The inferior parietal and superior temporal lobes participate in cognitive aspects of processing sensory stimuli, including pain. [52], [53] More specifically, both of these regions have been implicated in attentional orienting to environmental stimuli. [54], [55] In the study reported herein, the superior temporal/inferior parietal region for which there was a negative correlation with pain thresholds in controls but a lack of such correlation in migraineurs is estimated to be located within the ventral frontoparietal attention network. [56], [57] This network is activated when relevant stimuli are detected and is involved in orienting and re-orienting in response to the relevant stimulus. [55], [58] Although the ventral frontoparietal attention network is typically right hemisphere dominant, with attention-demanding tasks this lateralization is lost. [55], [58] Activation of the superior temporal/inferior parietal cortex during painful stimuli might allow for pain modulation via directing attention away from the painful stimuli. Distraction from pain has been shown to reduce pain-induced activations of brain pain processing regions and has been shown to reduce pain sensation. [59]

Abnormal structure and function of superior temporal and inferior parietal regions have previously been demonstrated in migraineurs. A voxel-based morphometry study found that migraineurs have lower gray matter volume in the right superior temporal gyrus with extension to the parietal operculum compared to controls. [60] A recent fMRI study in our lab showed controls to have greater pain-induced activation of a right superior temporal region compared to pain-free migraineurs. [19] An fMRI study of interictal migraine with aura patients demonstrated migraineurs to have greater visual stimuli-induced activation of inferior parietal lobule compared to controls. [61] Finally, migraineurs have been shown to have abnormal centrality of functional and structural connectivity networks involving a hub in the inferior parietal lobe. [62]

A lack of correlation between pain thresholds and superior temporal/inferior parietal cortex cortical thickness found in our study and the atypical structure and function of these regions found in other migraine studies could represent a lack of the normal attention orienting role of the superior temporal/inferior parietal cortex in pain processing and a resulting inability to adequately direct attention away from pain. Since distraction from painful stimuli is a powerful method of reducing the pain experience, inability to direct attention away from pain could associate with migraineurs having higher ratings of pain intensity and pain unpleasantness, somatosensory amplification, and hypervigilance. Prior work has shown that migraineurs have enhanced expectations for pain and hypervigilance regarding pain. [12], [13]

### Study Limitations

Since cortical thickness might be fluid, changing over time in response to pain and other factors, the cross-sectional design of our study is a limitation. Longitudinal studies that correlate changes in migraine patterns, pain thresholds, and cortical thickness would help to better define the direction of the relationship between cortical thickness, migraine, and pain thresholds. Although the cortical thickness of the superior temporal/inferior parietal region was numerically thinner in migraineurs compared to controls, there was no significant difference in cortical thickness of this region between subject cohorts. Cortical thickness differences between migraineurs and controls might be identified for this region if this study had a larger sample size and if the analysis accounted for age. [63] There were no significant differences in pain thresholds between the migraineurs and control subjects in this study. Prior studies have suggested that such a difference exists. [9], [11] Further investigations of pain thresholds in migraineurs are needed to delineate whether headache-free migraineurs have pain thresholds that differ from non-migraine healthy controls. Thus, atypical superior temporal/inferior parietal cortical thickness to pain threshold correlations in migraineurs did not associate with lower pain thresholds in the migraineurs compared to the controls. It is possible that the alteration in function that likely corresponds with atypical superior temporal/inferior parietal cortical thickness would correlate with pain intensity ratings and/or ratings of pain unpleasantness, measures not collected in this study. Future studies should investigate correlations between pain intensity ratings, pain unpleasantness, and superior temporal/inferior parietal cortical thickness in migraineurs.

## Conclusions

In this study, migraineurs had absence of the normal pain threshold-to-cortical thickness correlation in a region of the superior temporal and inferior parietal cortex. Since this region is typically involved in attention and orienting to sensory stimuli, loss of such a correlation might imply that the migraine brain has less ability to re-orient attention away from painful stimuli. Since distraction from painful stimuli lessens pain sensation, lack of ability to distract oneself from pain could lead to an enhancement of the pain experience for migraineurs.

### Data Accessibility Statement

Researchers wishing to access our data should send their request via e-mail to the corresponding author (schwedt.todd@mayo.edu) and the Mayo Clinic Institutional Review Board (IRBE@mayo.edu).
